# IL-6 and lL-17 as potential biomarkers for premature coronary artery disease: a cross-sectional study

**DOI:** 10.1186/s12872-025-05227-0

**Published:** 2026-01-20

**Authors:** Kexin Yang, Sheng Liu, Chenyang Wang, Siyao Ni, Zhijian Yue, Ludan Bi, Yunxiao Yang, Ming Zhang

**Affiliations:** 1 Center for Coronary Heart Disease, Beijing Anzhen Hospital, Capital Medical University, Beijing, China; 2 Center for Coronary Heart Disease, Beijing Anzhen Hospital, Capital Medical University, Yanqing Township Community Health Center, Yanqing District, Beijing, China

**Keywords:** Inflammation, Premature coronary artery disease (pCAD), IL-6, IL-17, Atherosclerosis

## Abstract

**Background:**

Coronary artery disease (CAD) is a chronic inflammatory condition, with cytokines playing a crucial role. However, their involvement in premature CAD (pCAD) remains unclear. This study investigates the relationship between cytokines and pCAD.

**Methods:**

A total of 986 patients (males ≤ 55 years, females ≤ 65 years) were classified into pCAD and non-CAD groups based on coronary angiography. Serum levels of 12 cytokines, including Interleukin–1β (IL–1β), IL-2, IL-4, IL-5, IL-6, IL-8, IL-10, IL-12p70, IL-17, tumor necrosis factor–α (TNF–α), interferon–α (IFN–α), and IFN-γ, were measured. Spearman correlation and binary logistic regression were used to evaluate the relationship between cytokines and pCAD. The predictive performance of the multivariable logistic regression model was evaluated using ROC curve analysis.

**Results:**

Serum levels of IL-6 (*P* < 0.001) and IL-17 (*P* = 0.048) were significantly higher in the pCAD group. In males, both IL-6 (*P* = 0.039) and IL-17 (*P* = 0.039) levels were higher compared to non-CAD, while in females, only IL-6 was significantly different (*P* = 0.008). Moreover, IL-6 showed moderate positive correlations with hsTnI (*r* = 0.490, *P* < 0.001) and hsCRP (*r* = 0.542, *P* < 0.001). IL-17 (OR = 1.042, 95% CI: 1.005–1.080, *P* = 0.026), hsTnI (OR = 1.038, 95% CI: 1.008–1.069, *P* = 0.014), diabetes (OR = 1.903, 95% CI: 1.160–3.121, *P* = 0.011), and aspirin use (OR = 1.766, 95% CI: 1.131–2.757, *P* = 0.010) were independent risk factors for pCAD. ROC analysis showed an AUC of 0.792 for the full model and 0.747 for significant predictors.

**Conclusion:**

IL-6 and IL-17 were significantly higher in the pCAD group. IL-6 was positively correlated with hsTnI and hsCRP. IL-17, hsTnI, diabetes, and aspirin use were independent risk factors, highlighting inflammation’s significance in pCAD.

**Supplementary Information:**

The online version contains supplementary material available at 10.1186/s12872-025-05227-0.

## Introduction

Coronary artery disease (CAD) is the leading cause of death worldwide [1]. With changes in modern lifestyles, the age of onset has gradually decreased, and the prevalence among younger populations continues to rise [2]. The National Cholesterol Education Program - Adult Treatment Panel III (NCEP-ATP III) defines premature coronary artery disease (pCAD) as the onset of CAD before the age of 55 in men and before the age of 65 in women [3]. Early-onset CAD is often insidious and asymptomatic in the initial stages but progresses rapidly, frequently presenting as acute coronary syndrome (ACS). It is associated with higher recurrence rates and mortality, posing a serious threat to younger patients [4]. Compared to late-onset CAD, pCAD has a greater impact on the patients’ quality of life and prognosis, placing a substantial economic burden on both families and society. Therefore, early detection, diagnosis, and intervention are of great importance.

Atherosclerosis (AS) is the primary pathological basis of CAD. Studies have shown that AS is not merely a process of lipid deposition, but rather a chronic inflammatory disease [5], where inflammation bridges lipids and other traditional risk factors to CAD. Various cytokines, including interleukins (IL), tumor necrosis factor-alpha (TNF-α), and interferon-gamma (IFN-γ), regulate inflammation by transmitting both pro-inflammatory and anti-inflammatory signals, thereby driving the progression of AS [6]. For instance, the CANVAS (Canagliflozin Cardiovascular Assessment Study) found that each doubling of IL-6 in circulation increased the risk of cardiovascular and renal outcomes by 15% [7]. Furthermore, a 25% reduction in IL-6 levels was associated with a 7% decrease in the risk of these outcomes [8]. Additionally, IL-17 is also linked to CAD progression. Increased IL-17 A levels have been observed in atherosclerotic plaques, and circulating IL-17 A levels are elevated in various cardiovascular diseases [9]. Therefore, cytokine levels could serve as indicators for assessing the severity and prognosis of CAD.

Based on the above, a clear correlation exists between cytokines and CAD; however, their role in pCAD—particularly potential gender-specific variations—remains unexplored. To address these gaps, this study aims to investigate the association of cytokines with pCAD in a gender-stratified manner, providing new insights for early diagnosis and personalized treatment strategies. The primary outcome was the comparison of plasma cytokine levels between pCAD patients and angiography-negative controls. Secondary outcomes included examining the associations of cytokine levels with conventional cardiovascular risk factors and evaluating their diagnostic value for pCAD, with analyses stratified by gender.

## Materials and methods

### Study population

**Fig. 1:**
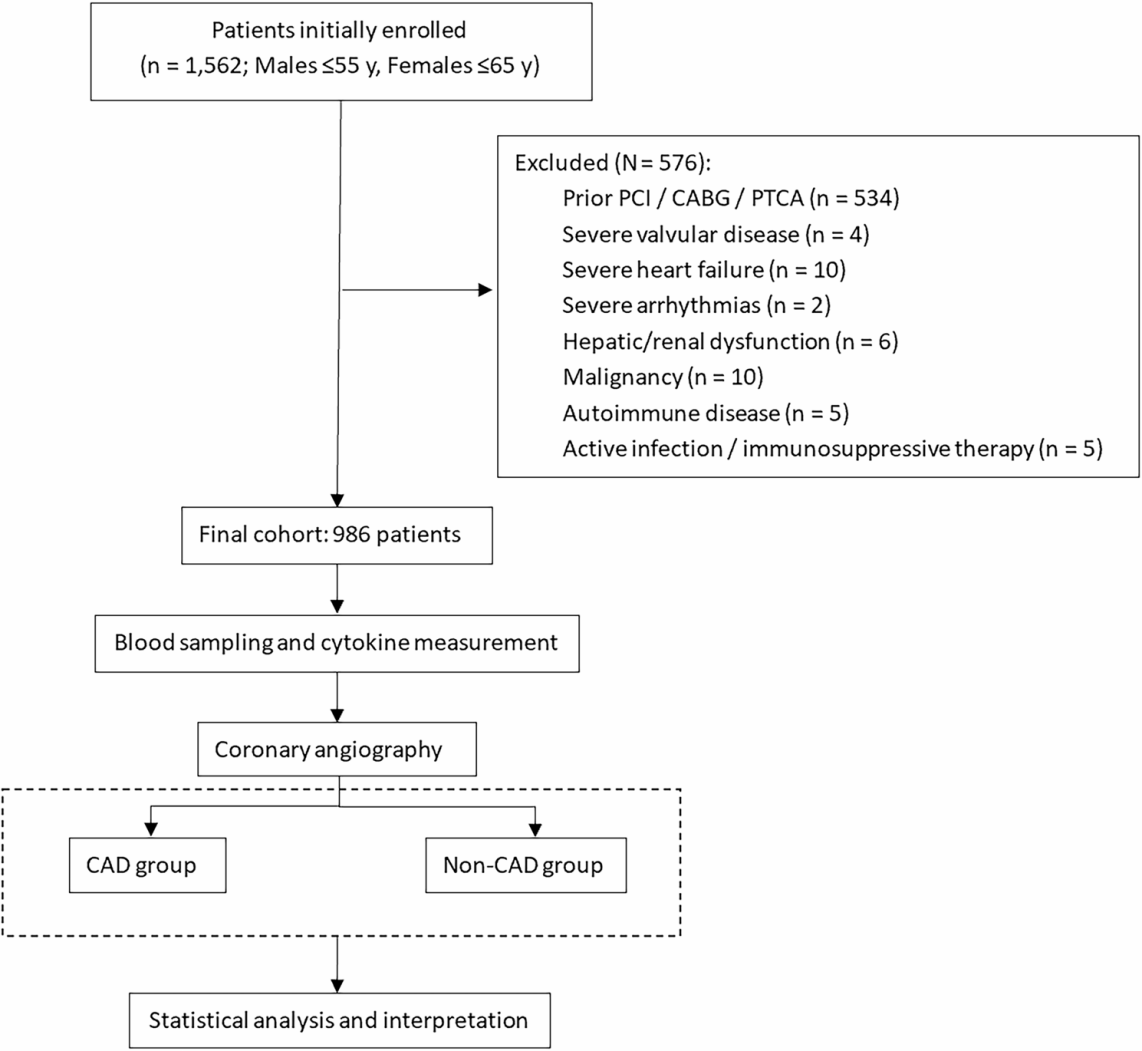
Study flow diagram. A total of 1,562 patients were initially enrolled, of whom 576 were excluded based on predefined criteria. The final cohort consisted of 986 patients who underwent CAG and were subsequently classified into CAD and non-CAD groups

This was a single-center, retrospective, cross-sectional study. From March 8, 2021, to November 10, 2023, a total of 1,562 patients (males ≤ 55 years, females ≤ 65 years) were initially enrolled from the Cardiovascular Medicine Department of Beijing Anzhen Hospital. Patients who had previously undergone coronary stenting, coronary artery bypass grafting, or balloon angioplasty were excluded (*n* = 534), leaving 1,028 patients. Additional exclusion criteria were applied: severe valvular heart disease (*n* = 4), severe heart failure (*n* = 10), severe arrhythmias (*n* = 2), significant hepatic or renal dysfunction (*n* = 6), malignancies (*n* = 10), autoimmune diseases (*n* = 5), active infections or use of anti-inflammatory drugs/immunosuppressive agents (*n* = 5), resulting in a final analytic cohort of 986 patients. All participants underwent percutaneous coronary angiography (CAG) based on clinical judgment. Referrals were made according to the 2019 ESC Chronic Coronary Syndrome (CCS) guideline framework of clinical likelihood [10]. A formal numerical pre-test probability (PTP) was not routinely calculated; referral decisions were based on age, sex, symptom characteristics, and available non-invasive test results (e.g., ECG, biomarker findings). A small subset of patients (*n* = 8) underwent angiography primarily due to patient preference. Patients were categorized into CAD and non-CAD groups based on CAG results. CAD was defined as obstructive disease, i.e., ≥ 50% diameter stenosis in at least one major coronary artery segment, as visually estimated on invasive CAG. This study was approved by the Medical Ethics Committee of Beijing Anzhen Hospital (Approval No. 2020025X) in accordance with the Declaration of Helsinki, under the supervision of Dr. Zhang Ming. Written informed consent was obtained from all participants. Venous blood samples were collected in the morning after ≥ 12 h of fasting, centrifuged at 2,500 g for 10 min, and serum was stored at − 80 °C for cytokine assays. The study design and patient selection process are illustrated in Fig. 1.

### Demographic and clinical information

The clinical characteristics of each patient were recorded, including gender, age, body mass index (BMI), hypertension, hyperlipidemia, diabetes mellitus, smoking history, and the use of aspirin, statins, angiotensin-converting enzyme inhibitors (ACEIs), angiotensin receptor blockers (ARBs), and beta-blockers. Biochemical markers measured included total triglycerides (TG), total cholesterol (TC), high-density lipoprotein cholesterol (HDL-C), low-density lipoprotein cholesterol (LDL-C), non-high-density lipoprotein cholesterol (non-HDL-C), high-sensitivity C-reactive protein (hsCRP), homocysteine (Hcy), estimated glomerular filtration rate (eGFR), urea, serum creatinine (Scr), fasting blood glucose (FBG), and high-sensitivity troponin I (hsTnI). These parameters were analyzed using a biochemical analyzer (Hitachi-7600, Tokyo, Japan) and a chemiluminescent immunoassay analyzer (Abbott-i2000SR, Illinois, USA). Quality control samples were processed in a blinded manner, with the intra-assay coefficient of variation < 5 and the inter-assay coefficient of variation < 10%.

### Cytokine assay

The concentrations of serum cytokines (IL-1β, IL-2, IL-4, IL-5, IL-6, IL-8, IL-10, IL-12p70, IL-17, TNF-α, IFN-α, IFN-γ) were measured using a human cytokine assay kit (Ruisikeer Biotechnology, Laoshan, Qingdao, China), employing multiplex particle-based flow cytometric assays technology on the FACSCanto II system (BD Biosciences, San Jose, CA, USA). Further analysis was conducted using FACSCanto clinical software (BD Biosciences, San Jose, CA, USA). All procedures were performed according to the manufacturer’s instructions. A standard curve was generated for each cytokine, and the linear portion of the curve was used to convert the mean fluorescence intensity to the concentration of each cytokine per well.

### Statistical analysis

Categorical variables were presented as frequencies or percentages (n%). Continuous variables with normal distribution were expressed as mean ± standard deviation, while non-normally distributed variables were presented as median (25th–75th percentile). Cases with missing data were excluded from the analyses, and no imputation was performed. The normality of continuous variables was assessed using the Shapiro–Wilk test. For comparisons between the non-CAD and pCAD groups, continuous variables were analyzed using the independent samples t-test or the Mann–Whitney U test, and categorical variables were analyzed using the chi-squared test. Spearman correlation analysis was conducted to assess the relationship between cytokines and risk factors for pCAD.

Variables that were statistically significant in univariate logistic regression (*P* < 0.05), together with variables considered clinically relevant, were included as candidate predictors in a multivariable binary logistic regression model to identify independent risk factors for pCAD. Multicollinearity among predictors was assessed using variance inflation factors (VIF), and variables with high VIF (> 10) were excluded.

Model discrimination was evaluated using ROC curve analysis: [1] using predicted probabilities from the full multivariable model including all candidate variables, and [2] using predicted probabilities calculated from only the variables that remained statistically significant in the multivariable model. The area under the ROC curve (AUC) with 95% confidence intervals was used to assess the predictive performance of the models. Model calibration was assessed using the Hosmer–Lemeshow (H-L) goodness-of-fit test.

To account for potential overfitting and to provide more robust estimates of model performance, we performed internal validation using bootstrap-based optimism correction. We generated 1,000 bootstrap samples from the original dataset and calculated the optimism, defined as the difference between the AUC in each bootstrap sample and the AUC obtained when applying the corresponding bootstrap-derived model to the original dataset. The optimism-corrected AUC was then calculated by subtracting the average optimism from the apparent (original) AUC.

## Results

### Characteristics of the study population

The baseline characteristics of the patients participating in this study are summarized in Table 1. Compared with the non-CAD group, the pCAD group had a significantly higher proportion of males (*P* < 0.001), as well as higher rates of smoking (*P* < 0.001), diabetes (*P* = 0.040), and aspirin use (*P* < 0.001). In addition, the pCAD group showed higher levels of TG (*P* < 0.001), hsCRP (*P* < 0.001), Hcy (*P* < 0.001), hsTnI (*P* < 0.001), eGFR (*P* = 0.023), and Scr (*P* < 0.001). In contrast, the non-CAD group had a greater average age (*P* < 0.001) and higher HDL-C levels (*P* < 0.001).

**Table 1:** Clinical characteristics and serum cytokine levels of patients in each group

Group	Non-CAD (*n* = 159)	CAD (*n* = 827)	*P*
BMI (kg/m2)	26.1 (23.4, 28.5)	26.6 (24.5, 29.4)	0.054
Age, years	54.0 (50.3, 57.0)	51.0 (44.0, 55.0)	< 0.001
Male, n (%)	72(45.3%)	590(71.3%)	< 0.001
Smoking, n (%)	35(22.0%)	353(42.7%)	< 0.001
Basal diseases, n (%)			
Hypertension	85(53.5%)	462(55.9%)	0.576
Hyperlipidemia	104(65.4%)	592(71.6%)	0.118
Diabetes	39(24.5%)	271(32.8%)	0.040
Medications, n (%)			
Aspirin	48(30.2%)	378(45.7%)	< 0.001
Statin	57(35.8%)	344(41.6%)	0.177
ACEI/ARB	15(9.4%)	85(10.3%)	0.747
Beta-blockers	7(4.4%)	41(5.0%)	0.766
Cytokines (pg/ml)			
IL-1β	1.63 (0.87, 2.92)	1.71 (0.91, 2.54)	0.744
IL-2	1.11 (0.60, 1.60)	1.22 (0.69, 1.75)	0.237
IL-4	1.41 (0.68, 1.97)	1.40 (0.80, 1.96)	0.790
IL-5	0.80 (0.44, 1.16)	0.79 (0.49, 1.15)	0.946
IL-6	3.76 (2.50, 5.07)	4.59 (2.93, 8.28)	< 0.001
IL-8	29.0 (17.0, 52.8)	30.3 (18.7, 54.2)	0.601
IL-10	2.42 (1.63, 3.26)	2.65 (1.90, 3.63)	0.162
IL-12p70	1.59 (0.89, 2.66)	1.74 (1.04, 2.71)	0.855
IL-17	4.91 (1.36, 10.57)	6.42 (2.48, 11.60)	0.048
TNF-α	1.60 (0.89, 2.08)	1.38 (0.76, 2.08)	0.140
IFN-α	1.24 (0.64, 2.06)	1.21 (0.72, 1.76)	0.522
IFN-γ	1.10 (0.64, 1.87)	1.08 (0.58, 1.79)	0.159
Biochemical parameters			
hsCRP (mg/l)	1.03 (0.47, 2.62)	1.64 (0.77, 4.83)	< 0.001
TG (mmol/L)	1.46 (1.14, 2.08)	1.78 (1.27, 2.66)	< 0.001
TC (mmol/L)	4.24 (3.58, 5.00)	4.29 (3.55, 5.07)	0.485
HDL–C (mmol/L)	1.13 (0.98, 1.34)	1.00 (0.87, 1.18)	< 0.001
LDL–C (mmol/L)	2.36 (1.74, 3.06)	2.45 (1.83, 3.18)	0.226
Urea (mmol/L)	5.18 (4.22, 6.00)	5.20 (4.34, 6.38)	0.107
Scr (µmol/L)	69.0 (59.1, 79.8)	72.3 (62.9, 82.2)	< 0.001
Hcy (µmol/L)	11.95 (9.70, 14.35)	12.85 (10.70, 16.25)	< 0.001
hsTnI (pg/ml)	2.60 (1.40, 4.00)	5.60 (2.30, 181.00)	< 0.001
eGFR (mL/min)	98.9 (90.4, 103.9)	101.1 (91.6, 108.1)	0.023

Values are presented as n (%), mean ± SD, or median (25th–75th percentile), as appropriate. Comparisons between CAD and non-CAD groups were performed using Student’s t-test or Mann–Whitney U test for continuous variables, and chi-square test for categorical variables

*P* < 0.05 was considered statistically significant

(Abbreviations: BMI, body mass index; ACEI, angiotensin-converting enzyme inhibitor; ARB, angiotensin receptor blocker; hsCRP, high-sensitivity C-reactive protein; TG, triglycerides; TC, total cholesterol; HDL–C, high-density lipoprotein cholesterol; LDL–C, low-density lipoprotein cholesterol; Scr, serum creatinine; Hcy, homocysteine; hsTnI, high-sensitivity troponin I; eGFR, estimated glomerular filtration rate; IL, interleukin; TNF-α, tumor necrosis factor-alpha; IFN-α, interferon-alpha)

### Serum cytokines levels across groups

**Fig. 2:**
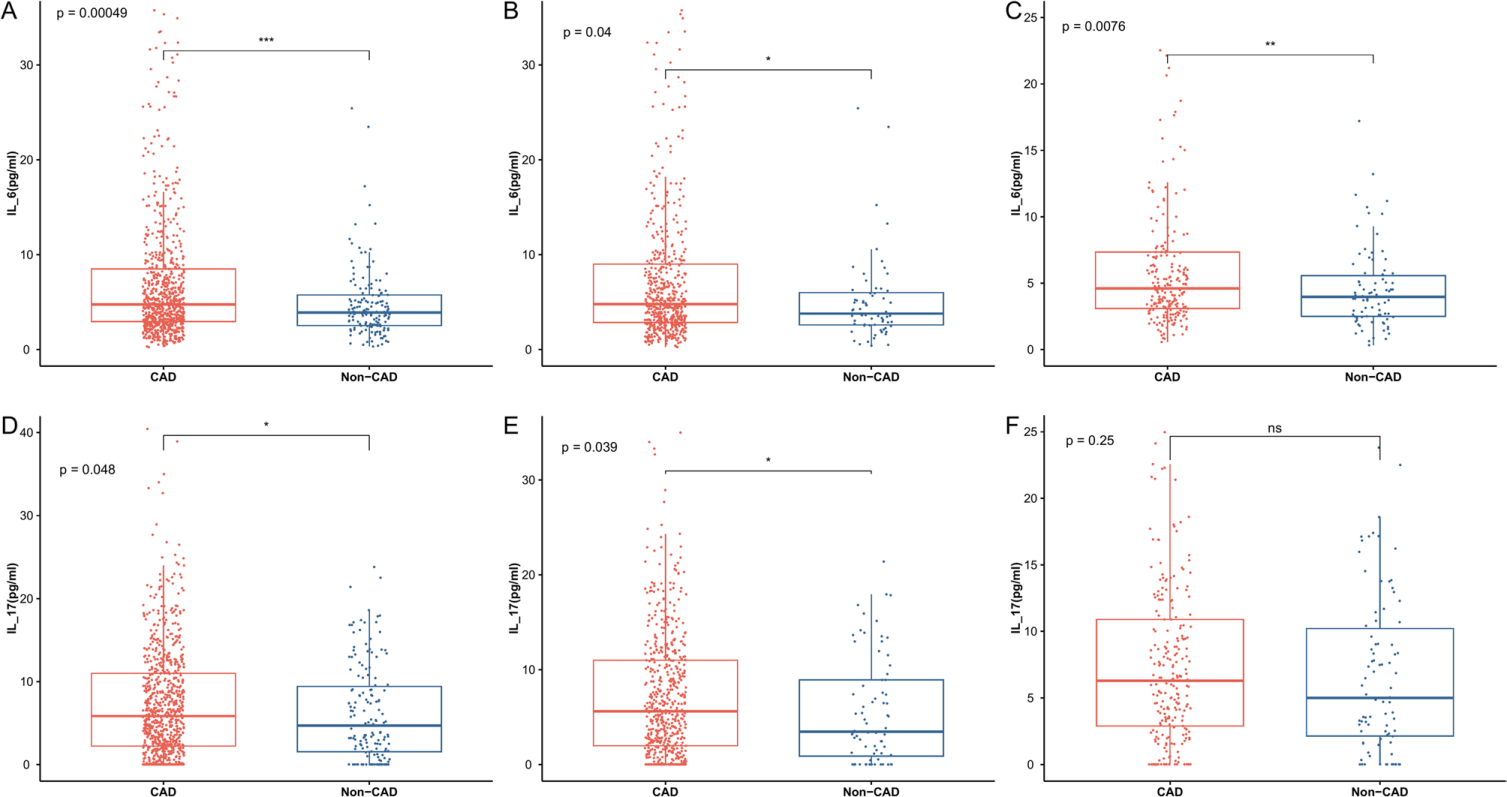
Data are expressed as medians with the 25th and 75th percentiles. Red represents the CAD group, and blue represents the non-CAD group. Row 1 **A**, **B**, **C** compares IL-6 levels between CAD and non-CAD groups across the overall population, male subgroup, and female subgroup, respectively. Row 2 (D, E, F) shows a similar comparison of IL-17 levels between CAD and non-CAD patients in the corresponding subgroups *: *P* < 0.05, **: *P* < 0.01, ***: *P* < 0.001, ns: no significant difference (Abbreviations: IL-17, interleukin-17; IL-6, interleukin-6)

The results of serum cytokine measurements are shown in Table 1. No statistically significant differences were observed in the levels of cytokines between the pCAD and non-CAD groups, except for IL-6 and IL-17. Fig.2 , illustrates the levels of IL-6 and IL-17 in each group. Compared to the non-CAD group, IL-6 (*P* < 0.001) and IL-17 (*P* = 0.048) levels were significantly higher in the pCAD group. When analyzed by sex, male patients in the pCAD group had significantly higher IL-6 (*P* = 0.040) and IL-17 (*P* = 0.039) levels than male non-CAD patients. In the female group, only IL-6 levels differed significantly between the pCAD and non-CAD groups (*P* = 0.008), with no significant difference in IL-17 levels.

### Correlation between serum cytokines and clinical parameters

**Fig. 3:**
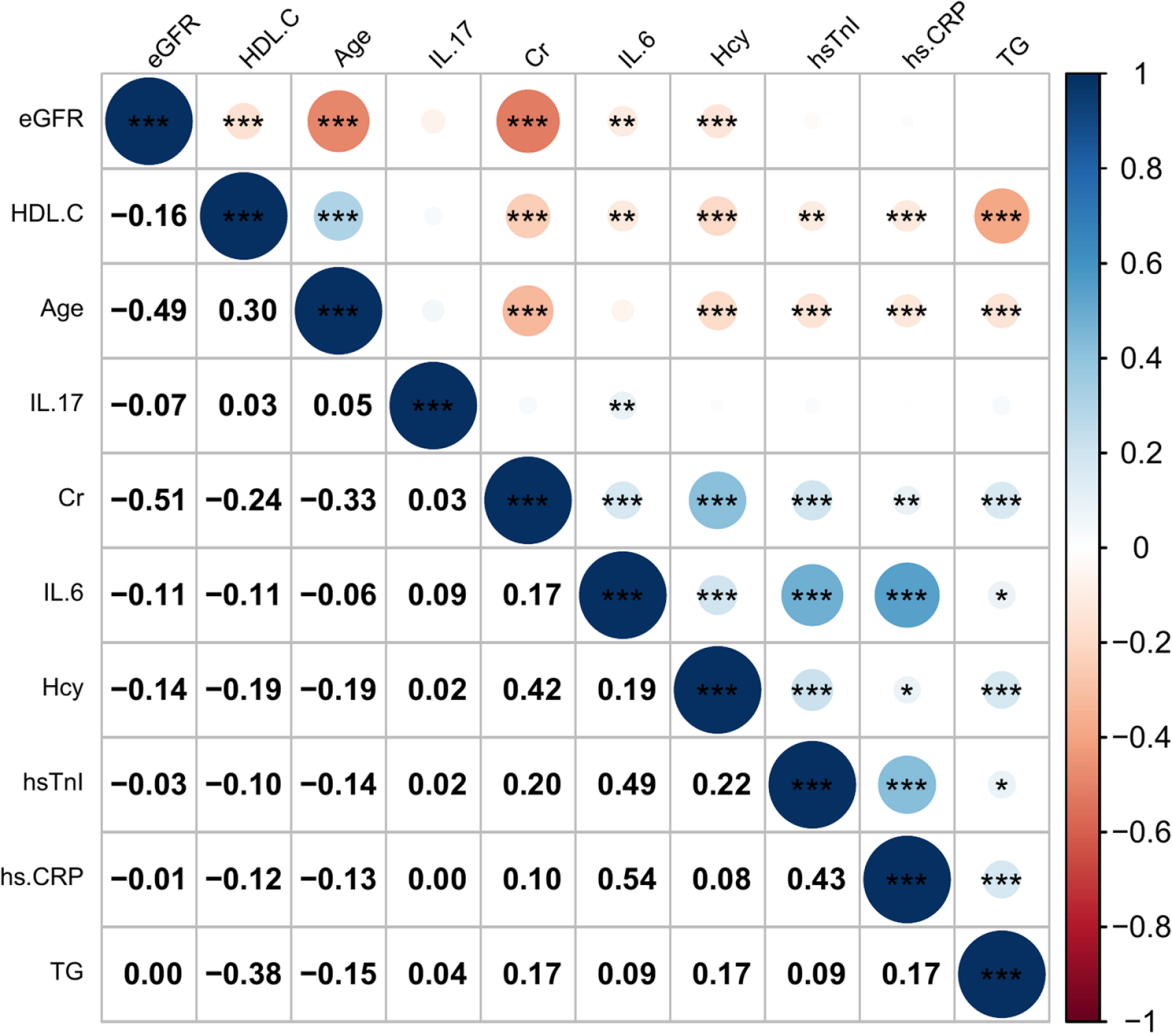
Spearman correlation analysis of variables with statistically significant differences between the non-CAD and CAD groups was performed. The larger the area of the circle, the greater the absolute value of the correlation coefficient. The color indicates the direction and strength of the correlation, with blue representing positive correlations and red representing negative correlations; deeper colors correspond to stronger correlations. Numerical values on the color scale range from − 1 (strong negative correlation) to + 1 (strong positive correlation) *P < 0.05, **P < 0.01, ***P < 0.001 (Abbreviations: IL-17, interleukin-17; IL-6, interleukin-6; hsCRP, high-sensitivity C-reactive protein; TG, triglycerides; HDL-C, high-density lipoprotein cholesterol; Cr, serum creatinine; Hcy, homocysteine; hsTnI, high-sensitivity troponin I; eGFR, estimated glomerular filtration rate)

**Fig. 4:**
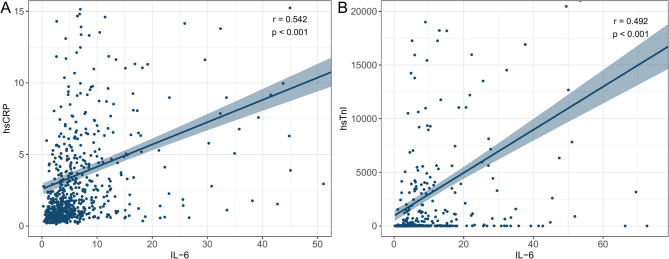
Scatterplots of hsCRP, hsTnI, and IL-6 levels. r, correlation coefficient. A and B show the correlations between hsCRP and IL-6, and hsTnI and IL-6, respectively. *P* < 0.05 was considered statistically significant (Abbreviations: hsCRP, high-sensitivity C-reactive protein; hsTnI, high-sensitivity troponin I)

As shown in Fig. 3, Spearman correlation analysis was used to assess the relationship between serum cytokines and other parameters with statistically significant differences. Among them, hsTnI (*r* = 0.490, *P* < 0.001) and hsCRP (*r* = 0.542, *P* < 0.001) showed moderate positive correlations with IL-6. The results mentioned above are presented in Fig. 4. Other parameters, such as HDL-C (*r* = −0.113, *P* = 0.001), IL-17 (*r* = 0.091, *P* = 0.009), Cr (*r* = 0.171, *P* < 0.001), and Hcy (*r* = 0.193, *P* < 0.001), also exhibited statistically significant correlations with IL-6, but the correlations were weaker.

### Multivariable logistic regression analysis and ROC assessment

To identify the independent risk factors for pCAD, we conducted multivariate binary logistic regression analysis to assess the relationship between various parameters and pCAD. Multicollinearity among candidate predictors was assessed using VIF; Scr was excluded from the multivariable model due to a high VIF (26.24), and the remaining predictors had acceptable VIF values (maximum = 1.97). As shown in Table 2, IL-17 (OR = 1.042, 95% CI: 1.005–1.080, *P* = 0.026), hsTnI (OR = 1.038, 95% CI: 1.008–1.069, *P* = 0.014), aspirin use (OR = 1.766, 95% CI: 1.131–2.757, *P* = 0.010), and diabetes (OR = 1.903, 95% CI: 1.160–3.121, *P* = 0.011) were significantly associated with pCAD.

**Table 2:** Logistic regression analysis for predicting pCAD

Variables	OR (95%CI)	*P*
Age	1.002 (0.965–1.040)	0.927
IL-17	1.042 (1.005–1.080)	**0.026**
IL-6	0.990 (0.971–1.010)	0.296
hs-CRP	1.031 (0.970–1.096)	0.325
TG	1.213 (0.962–1.530)	0.102
HDL-C	0.767 (0.326–1.804)	0.543
Hcy	1.018 (0.986–1.051)	0.276
hsTnI	1.038 (1.008–1.069)	**0.014**
eGFR	1.010 (0.992–1.028)	0.293
Male vs. Female	1.453 (0.809–2.611)	0.210
Smoking	1.584 (0.922–2.722)	0.095
Diabetes	1.903 (1.160–3.121)	**0.011**
Aspirin	1.766 (1.131–2.757)	**0.010**

The dependent variable in the logistic regression analysis is the presence of pCAD, and the independent variables are age, IL-17, IL-6, hs-CRP, TG, HDL-C, Hcy, hsTnI, eGFR, Male vs. Female, Smoking, Diabetes, and Aspirin use. Odds ratios (ORs) with 95% confidence intervals (CIs) are reported

*P* < 0.05 was considered statistically significant

(Abbreviations: IL-17, interleukin-17; IL-6, interleukin-6; hsCRP, high-sensitivity C-reactive protein; TG, triglycerides; HDL-C, high-density lipoprotein cholesterol; Hcy, homocysteine; hsTnI, high-sensitivity troponin I; eGFR, estimated glomerular filtration rate; pCAD, premature coronary artery disease.)

**Fig. 5:**
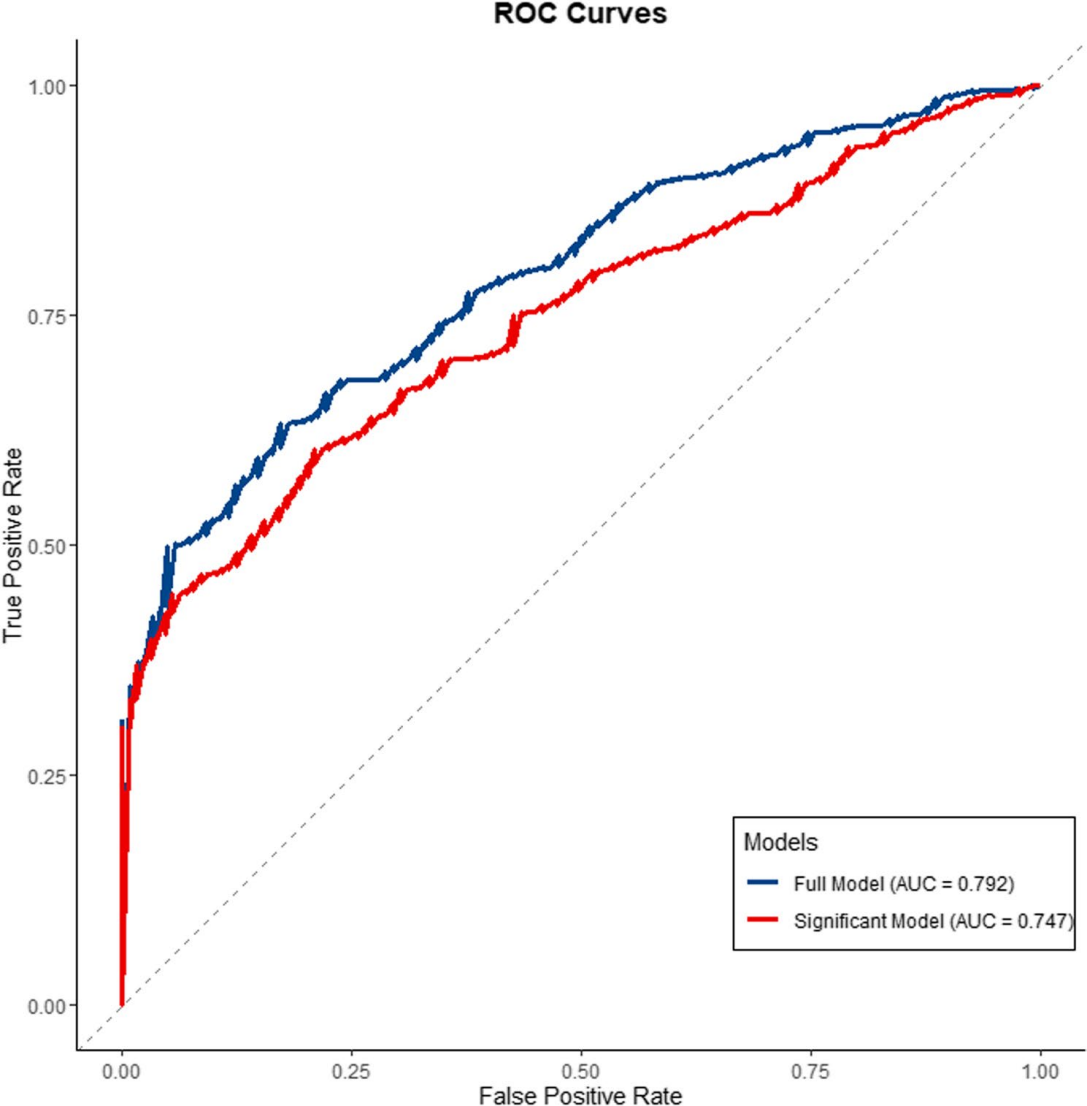
ROC curves of the multivariable logistic regression model. The blue curve represents the full model, generated using predicted probabilities from all candidate variables, while the red curve represents the model using only the variables that were statistically significant in the multivariable analysis (IL-17, hsTnI, aspirin use, diabetes). (Abbreviations: ROC, receiver operating characteristic; AUC, area under the curve; IL-17, interleukin-17; hsTnI, high-sensitivity troponin I)

The predictive ability of the model was evaluated using ROC curve analysis (Fig. 5). The full model, including all candidate variables, achieved an AUC of 0.792 (95% CI: 0.755–0.829), indicating good discrimination. When only the variables that remained statistically significant in the multivariable model were considered, the AUC was 0.747 (95% CI: 0.709–0.785), showing that these key predictors captured most of the model’s discriminative capacity.

To account for potential overfitting, internal validation used bootstrap optimism correction with 1,000 resamples. The optimism-corrected AUCs were 0.763 for the full model and 0.737 for the parsimonious model (Supplementary Figure S1), indicating that both models retained good discriminatory performance after internal validation. Detailed bootstrap results are provided in Supplementary Table S1.

**Fig. 6:**
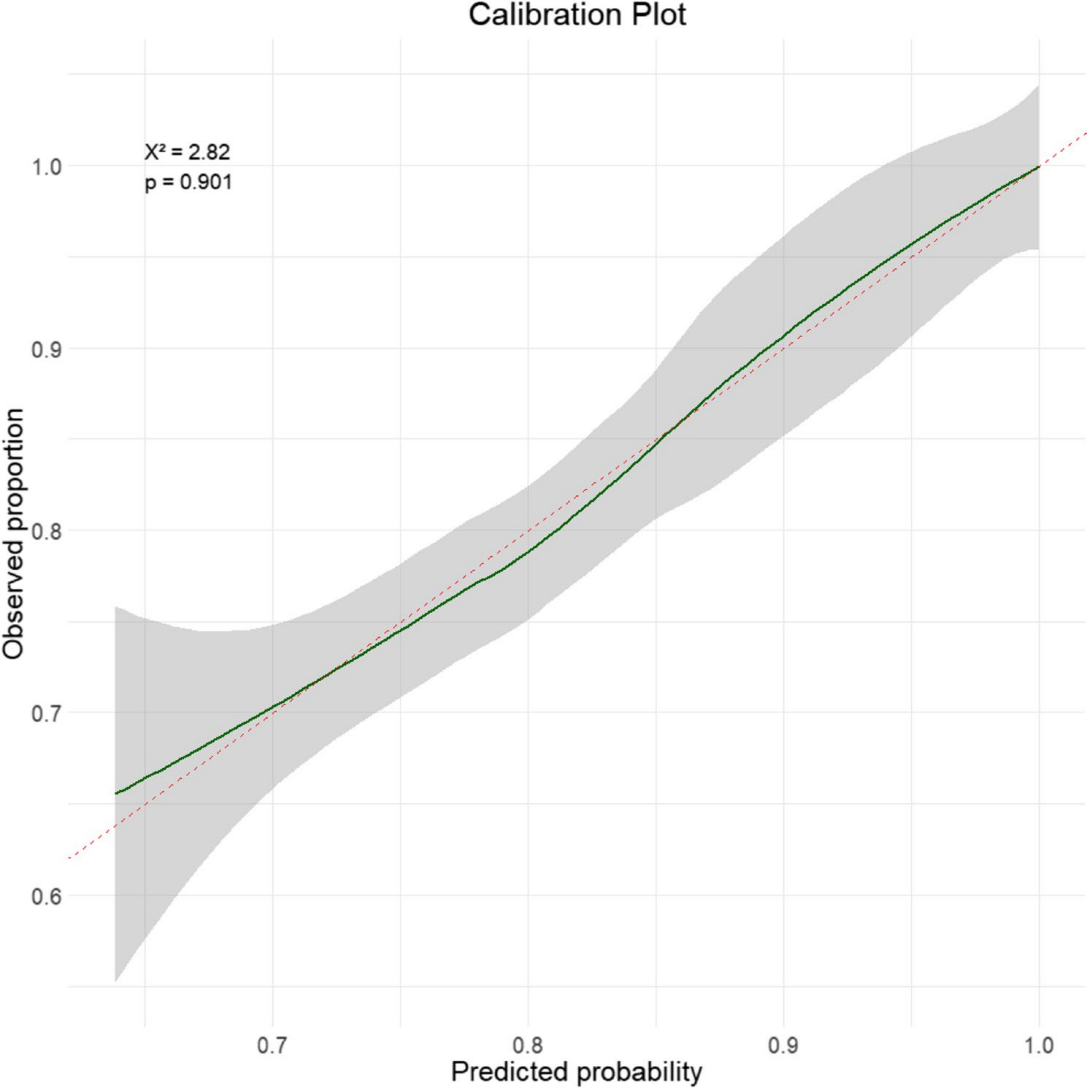
Calibration plot of the final multivariable logistic regression model. The green line shows the LOESS smoothed curve of observed proportions versus predicted probabilities, reflecting the model’s calibration. The red dashed line represents the ideal 45° diagonal line, indicating perfect agreement between predicted and observed outcomes. Hosmer-Lemeshow goodness-of-fit test: X² = 2.82, *p* = 0.901

Model calibration was assessed using the H-L goodness-of-fit test, which yielded X² = 2.82, df = 7, *P* = 0.901, indicating good fit. Calibration curves were plotted using locally weighted scatterplot smoothing (LOESS) to visually evaluate agreement between predicted probabilities and observed outcomes (Fig. 6).

## Discussion

CAD is a leading cause of death worldwide, with its increasing prevalence and mortality posing a significant threat to public health. The definition of pCAD varies, typically referring to CAD that occurs in men ≤ 55 years and women ≤ 65 years [11]. Although the incidence of pCAD has traditionally been low, changes in lifestyle, diet, and work patterns, along with increasing cardiovascular risk factors such as smoking and obesity among younger populations [12, 13], have contributed to a rising incidence of pCAD in recent years [14]. According to the Global Burden of Disease (GBD) 2019 study, early-onset ischemic heart disease (defined as occurring in men < 55 years and women < 65 years) accounted for approximately 1.44 million deaths worldwide in 2019, with an age-standardized mortality rate of 38.8 per 100,000 population [15]. Given its prolonged course, rapid progression, and the increased likelihood of major adverse cardiovascular events (MACE) in its early stages [16], investigating the factors related to the onset and progression of pCAD is of significant clinical importance.

Our study indicates that serum cytokine levels are associated with the occurrence of pCAD. Compared to the non-CAD group, serum levels of IL-6 and IL-17 were significantly higher in pCAD patients, and this difference remained in the male subgroup. However, in the female subgroup, only the IL-6 levels showed a significant difference. Spearman correlation analysis revealed varying degrees of positive correlation between hsCRP, hsTnI, and IL-6, suggesting that these factors may interact in the pathogenesis of pCAD. Multivariate logistic regression analysis further identified IL-17, hsTnI, and diabetes as independent risk factors for pCAD.

IL-6 is a multifunctional protein [17] that belongs to the type I cytokine family within the 4α-helix cytokine group. It promotes inflammation through endothelial activation and stimulation of fibrinogen synthesis [18]. Studies have shown that IL-6 is associated with the development of various inflammatory diseases, such as systemic lupus erythematosus [19], systemic sclerosis [20], and inflammatory myopathy [21]. Its role in AS has been studied in both animal models and human studies. Seino et al. (1994) reported that the expression level of IL-6 mRNA in atherosclerotic vessels is 10 to 40 times higher than in non-atherosclerotic vessels [22]. Furthermore, high serum levels of IL-6 have been shown to predict an increased risk of MACE in patients with stable coronary heart disease [23]. Research by Mohammad Haji Aghajani et al. reported significantly elevated IL-6 levels in the serum of pCAD patients [24], and a study by Rosalinda Posadas-Sánchez et al. further confirmed this finding [25]. Our data also show that IL-6 levels are significantly higher in pCAD patients compared to the non-CAD group, consistent with the aforementioned studies, suggesting that IL-6 may also play a key role in linking inflammation to AS in younger patients. However, in our multivariate logistic regression analysis, IL-6 was not retained as an independent predictor of pCAD. This may reflect its strong correlation with other inflammatory markers, particularly hsCRP. The effect of IL-6 could therefore be synergistic with or masked by hsCRP, which appears to be a more robust indicator of systemic inflammation in our model. Thus, although IL-6 levels were clearly elevated in pCAD patients, its independent predictive value may be limited.

IL-17, a key pro-inflammatory cytokine, belongs to the IL-17 family (IL-17 A to IL-17 F) [26]. It is primarily secreted by Th17 cells and γδ-T cells [27]. By binding to IL-17 receptor (IL-17R) on target cells, IL-17 activates signaling pathways such as NF-κB, thereby promoting the recruitment of monocytes and neutrophils to sites of inflammation [28]. In AS, IL-17 is strongly associated with thrombosis and plaque instability [29]. Our study demonstrates that serum IL-17 levels are significantly higher in pCAD patients compared to those in the non-CAD group, a finding consistent with several previous studies. For instance, Salar Mahmoudi-Nejad et al. (2022) reported significantly higher IL-17R gene expression in the pCAD patient group than in the control group [30], suggesting an upregulation of IL-17R in AS. Similarly, Christian Erbel et al. (2011) observed increased IL-17 A levels in atherosclerotic plaques [31], while higher circulating IL-17 A levels have been reported in various cardiovascular diseases [9]. Chang et al. further demonstrated that IL-17 A promotes vascular calcification in human coronary smooth muscle cells [32]. Collectively, these findings strongly support the critical role of IL-17 in the pathogenesis of pCAD. Moreover, low methylation of the IL-17 A gene promoter region, closely linked to elevated IL-17 A expression in pCAD patients, may contribute to the development and progression of pCAD [26], suggesting that this epigenetic alteration may be a key contributor to the development and progression of pCAD. However, some studies have not identified a significant association between IL-17 and early-onset coronary artery disease [33], which may be attributable to ethnic differences or variations in study design. Importantly, although IL-17 was identified as an independent risk factor in our analysis, the effect size was modest (OR = 1.042). This suggests that, while IL-17 contributes to disease risk, its clinical relevance may lie less in isolation and more as part of a broader pro-inflammatory network.

The age criteria for pCAD (≤ 55 years for men, ≤ 65 years for women) were based on commonly accepted definitions in previous studies and may introduce minor bias in sex-specific comparisons. Compared to the non-CAD group, serum levels of IL-6 and IL-17 were significantly higher in pCAD patients, especially in male patients. However, among female patients, while IL-6 levels were higher, no significant difference in IL-17 levels was observed. This gender difference may be related to the immune-regulatory effects of estrogen. Previous studies have demonstrated that estrogen stabilizes the fibrous cap and reduces the risk of plaque rupture by inhibiting IL-17 production [34]. Specifically, estradiol indirectly suppresses IL-17 production in γδ-T cells by downregulating IL-1β levels, thereby modulating the inflammatory response^33^. After menopause, declining estrogen levels are associated with an increased burden of atherosclerosis. This decline also correlates with a heightened risk of plaque instability and rupture [34]. Recent evidence indicates that men are more likely to develop lipid-rich, rupture-prone plaques, whereas women more often present with plaque erosion, underscoring fundamental sex-related differences in CAD pathophysiology [35]. Our results support the important role of estrogen in gender-related atherosclerosis development. Nevertheless, we did not measure sex hormone levels or menopausal status in our cohort, which limits our ability to directly link endocrine status with cytokine changes. Future studies incorporating hormonal and menopausal assessments are warranted to provide a deeper understanding of the biological mechanisms underlying the observed gender differences.

Spearman correlation analysis suggested that IL-6 and IL-17 are involved in the metabolic and inflammatory networks related to pCAD. This aligns with recent evidence that mitochondrial-derived peptides, such as humanin, exert antioxidative, anti-inflammatory, and antiapoptotic effects essential for cardiovascular protection [36]. Specifically, IL-6 was moderately positively correlated with hsTnI and hsCRP, suggesting its involvement in the pathogenesis and progression of ASCVD (Atherosclerotic Cardiovascular Disease). Additionally, IL-6 showed a weak positive correlation with Cr, indicating a potential link between higher IL-6 levels and mild renal impairment. Studies have demonstrated that IL-6 promotes endothelial dysfunction and renal tubular interstitial damage, which may further exacerbate kidney function decline [37, 38]. Despite the weak correlation, our findings support the theory that inflammation plays a significant role in the association between ASCVD and kidney disease. Meanwhile, IL-17 showed weaker correlations with IL-6 and hsCRP, indicating that its inflammatory effects may operate through an independent mechanism. Moreover, the weak correlation of IL-17 in this study may indicate that it is more involved in local inflammatory responses rather than the regulation of systemic inflammation.

Multivariate logistic regression identified IL-17, hsTnI, diabetes, and aspirin use as independent risk factors for pCAD. The association with aspirin should be interpreted cautiously, as it likely reflects confounding by indication. Patients with suspected CAD or higher cardiovascular risk are more likely to receive aspirin therapy, which may account for this finding. Because data on the timing and indication of aspirin initiation were unavailable, we could not perform the suggested sensitivity analysis. This limitation underscores the need for careful interpretation and for future studies with more detailed medication data.

Our models showed good discriminatory performance (AUCs 0.747–0.792) with stable results after bootstrap validation, suggesting limited risk of overfitting. Notably, the parsimonious model that included only statistically significant predictors preserved most of the discriminative capacity of the full model, indicating that these variables capture the essential risk information. In addition, calibration analysis confirmed good agreement between predicted probabilities and observed outcomes. These findings support the robustness and clinical applicability of our model, and highlight the potential utility of a simplified predictor set for practical risk stratification in patients with suspected pCAD.

### Limitations

This study has several limitations that should be considered when interpreting the results. First, the study was designed as a single-center study with a homogeneous cohort, which may introduce regional bias. Second, there were baseline differences between the study groups, such as age, sex, and aspirin use. These factors and other relevant comorbidities were adjusted for in the multivariable logistic regression, and multicollinearity was checked using VIF. Residual confounding may still exist. Moreover, previous studies have shown that pCAD is closely related to genetic factors, but the genetic background was not assessed in this study, which may have overlooked the contribution of genetic susceptibility to the onset and progression of the disease. Fourth, functional assessment of coronary stenosis using FFR/iFR or intravascular imaging (OCT/IVUS) was not performed, and treatment decisions were based on angiography and clinical judgment, potentially introducing bias in lesion severity evaluation. Fifth, data on the results of non-invasive tests prior to CAG were not systematically collected; therefore, the proportion of patients with positive non-invasive findings could not be reported. Only symptomatic patients were included, whereas some individuals with pCAD may remain asymptomatic, which may limit the generalizability of our findings. Finally, the cross-sectional design of this study limits our ability to infer causal relationships between cytokine levels and pCAD. Therefore, while we identified associations, we cannot conclude that elevated IL-6 or IL-17 directly contribute to the development of pCAD. Future large-scale prospective studies are needed to further clarify the role of inflammatory factors in the diagnosis, risk stratification, and treatment of pCAD.

## Conclusion

Serum levels of IL-6 and IL-17 are significantly higher in patients with pCAD, especially in males, where the differences in both cytokines are more pronounced. Estrogen may explain the gender differences in immune regulation by inhibiting IL-17 production. IL-6 correlates with hsTnI, hsCRP, and creatinine levels, supporting its role in ASCVD. IL-17 and hsTnI are identified as independent risk factors for pCAD, providing valuable insights for early identification and targeted therapy of pCAD.

## Supplementary Information

Supplementary material 1.

## Data Availability

The datasets presented in this article are not readily available because of the protection of personal data and privacy restrictions. Requests to access the datasets should be directed to MZ (Email: [zhangming2279@hotmail.com](mailto: zhangming2279@hotmail.com)).

## Abbreviations

CAD: Coronary artery disease
PCAD: premature coronary artery disease
NCEP-ATP III: The national cholesterol education program - adult treatment Panel III
ACS: Acute coronary syndrome
AS: Atherosclerosis
IL: Interleukin
TNF-α: Tumor necrosis factor-α
IFN: Interferon
CANVAS: Canagliflozin cardiovascular assessment study
CAG: Coronary angiography
CCS: Chronic coronary syndrome
PTP: Pre-test probability
BMI: Body mass index
ACEIs: Angiotensin-converting enzyme inhibitors
ARBs: Angiotensin receptor blockers
TG: Triglycerides
TC: Total cholesterol
HDL-C: High-density lipoprotein cholesterol
LDL-C: Low-density lipoprotein cholesterol
Non-HDL-C: Non-high-density lipoprotein cholesterol
hSCRP: High-sensitivity C-reactive protein
HCY: Homocysteine
EGFR: Estimated glomerular filtration rate
SCR: Serum creatinine
FBG: Fasting blood glucose
HSTNI: High-sensitivity troponin I
MACE: Major adverse cardiovascular events
VIF: Variance inflation factors
AUC: The area under the ROC curve
H-L: Hosmer–lemeshow
LOESS: Locally weighted scatterplot smoothing
GBD: Global burden of disease
ASCVD: Atherosclerotic cardiovascular disease
