# Supplementary material for: IL-6 and lL-17 as potential biomarkers for premature coronary artery disease: a cross-sectional study
Source: BMC Cardiovasc Disord. 2026 Jan 20;26:150. doi: 10.1186/s12872-025-05227-0 (PMC12903338; doi:10.1186/s12872-025-05227-0)
Supplement: Supplementary file 1 — Supplementary material 1. [file 12872_2025_5227_MOESM1_ESM.docx]

Supplementary Material

1. **Supplementary Table S1.** Results of bootstrap internal validation for the logistic regression models. – Pages 2.

2. **Supplementary Figure S1.** Distribution of AUC values from bootstrap internal validation. – Pages 3.

**Detailed Bootstrap Validation Results (n = 1,000 iterations)**

| Statistic | Full Model | Parsimonious Model |
| --- | --- | --- |
| Apparent AUC | 0.792 | 0.747 |
| Optimism | 0.029 | 0.010 |
| Optimism-Corrected AUC | 0.763 | 0.737 |

**Supplementary Table S1.** Results of bootstrap internal validation for the logistic regression models. The table presents the apparent AUC (performance on the original dataset), average optimism (mean difference between bootstrap sample AUC and original dataset AUC across 1,000 resamples), and optimism-corrected AUC (estimated performance on new data) for both the full model including all candidate variables and the parsimonious model including only statistically significant predictors.


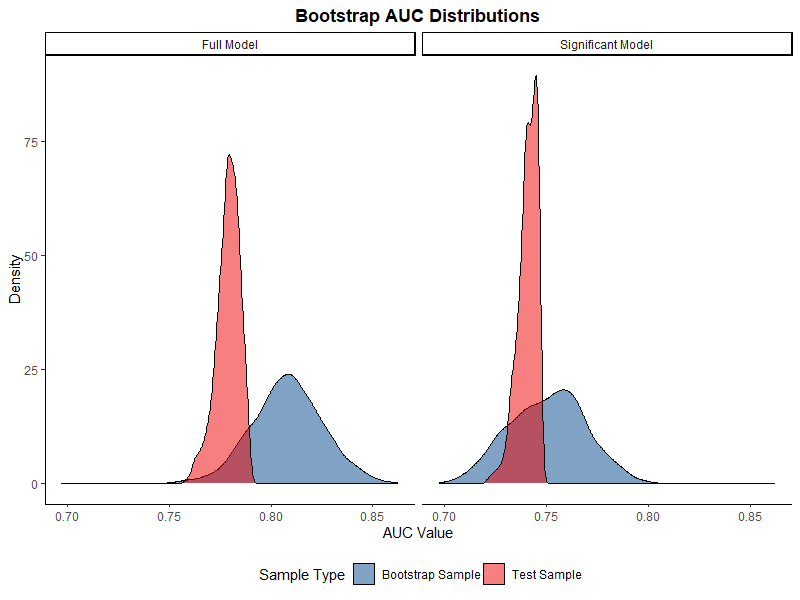


**Supplementary Figure S1.** Distribution of AUC values from bootstrap internal validation. Density plots show the distribution of AUC values across 1,000 bootstrap iterations for both the full model (left) and parsimonious model (right). The "Bootstrap Sample" curve (blue) represents AUC values when models are applied to the bootstrap samples themselves, while the "Test Sample" curve (red) shows AUC values when bootstrap-derived models are applied to the original dataset.
